# Three-Staged Surgical Hybrid Treatment for an Extended Thoracoabdominal Aneurysm in a High-Risk Patient: A Case Report

**DOI:** 10.7759/cureus.76616

**Published:** 2024-12-30

**Authors:** Shuji Setozaki, Hideyuki Katayama, Shuntaro Shimomura, Akira Takeuchi, Hiroshi Tsuneyoshi

**Affiliations:** 1 Department of Cardiovascular Surgery, Shizuoka General Hospital, Shizuoka, JPN; 2 Department of Cardiovascular Surgery, Numazu City Hospital, Shizuoka, JPN

**Keywords:** endovascular taaa repair, hybrid taaa repair, spinal cord ischemia, staged aortic repair, thoracoabdominal aortic aneurysm repair

## Abstract

Thoracoabdominal aortic aneurysm (TAAA) repair remains one of the most challenging procedures and is associated with high mortality and complication rates. Careful consideration of the surgical strategy is essential, particularly in cases involving extensive replacement and high-risk patients. A 61-year-old man with a 55-mm TAAA was referred for surgical treatment. Seven years earlier, he had undergone artificial vascular replacement for an infrarenal abdominal aortic aneurysm. His medical history included a myocardial infarction with a left ventricular ejection fraction (LVEF) of 20% and an apical thrombus. Additionally, he had chronic kidney disease, cerebral infarction, angina pectoris, hypertension, dyslipidemia, diabetes mellitus, and hyperuricemia. The preoperative plan involved a staged approach, beginning with total arch replacement (TAR) using the frozen elephant trunk (FET) technique and coronary artery bypass grafting (CABG), followed by thoracic endovascular aortic repair (TEVAR) and hybrid repair with abdominal debranching. The patient was discharged without complications. Although thoracoabdominal aortic surgery carries a high risk of mortality, it can be performed safely with careful preoperative assessment and the development of an individualized surgical strategy.

## Introduction

Thoracoabdominal aortic aneurysms (TAAAs) account for approximately 10% of all aortic aneurysms [1]. Patients with TAAAs have a very poor prognosis without surgical repair [2]. However, thoracoabdominal aortic surgery remains a challenging procedure [3], with more complex and extensive operations associated with even higher mortality and complication rates. For decades, open surgical repair has been the gold standard [4]. Recently, however, developments in endovascular and hybrid repair techniques have shown improved complication rates and reasonable durability. Each technique has its advantages and disadvantages, making it essential to optimize surgical and perioperative strategies based on the individual patient’s anatomy and comorbidities [5]. In this report, we describe a hybrid total thoracoabdominal aortic replacement performed in a high-risk patient with extensive TAAAs, achieving excellent results.

## Case presentation

**Figure 1:**
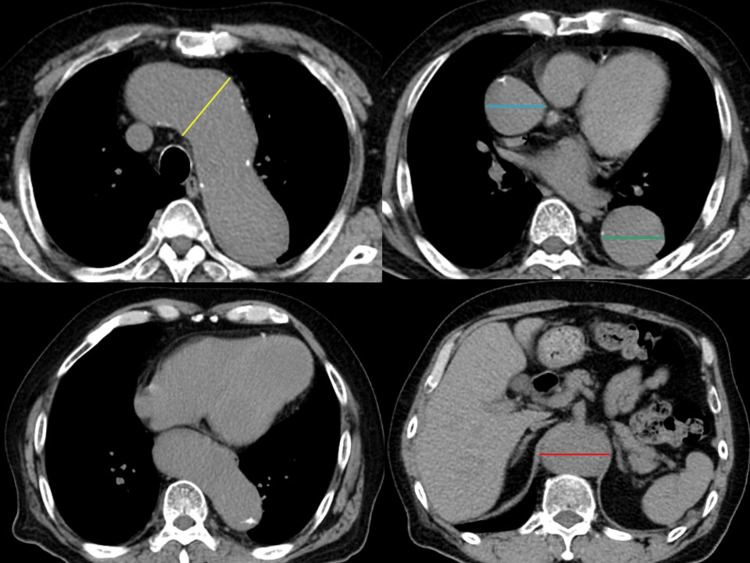
Preoperative plain computed tomography (CT) shows an extensive aortic aneurysm with a thoracoabdominal aneurysm CT showed that the ascending arch, descending aorta, and thoracoabdominal aorta measured 46 mm (blue line), 53 mm (yellow line), 43 mm (green line), and 55 mm (red line), respectively.

A 61-year-old man was referred to our hospital on September 16, 2021, because of a TAAA that was 55 mm in size, with a tendency to enlarge identified by computed tomography. Seven years ago, he had a 55 mm infrarenal abdominal aortic aneurysm and underwent abdominal aortic replacement. He had an old myocardial infarction with a markedly decreased LVEF of 20%, a history of apical thrombus, and was on warfarin. The electrocardiogram showed sinus rhythm. He also had chronic kidney disease (estimated glomerular filtration rate 30 ml/min/1.73 m^2^), an old cerebral infarction, stable angina pectoris, hypertension, dyslipidemia, diabetes mellitus, and hyperuricemia. Preoperative plain computed tomography (CT) showed that the ascending aorta was 46 mm, the arch aorta 53 mm, the descending aorta 43 mm, and the thoracoabdominal aorta 55 mm (Figure 1).

**Figure 2:**
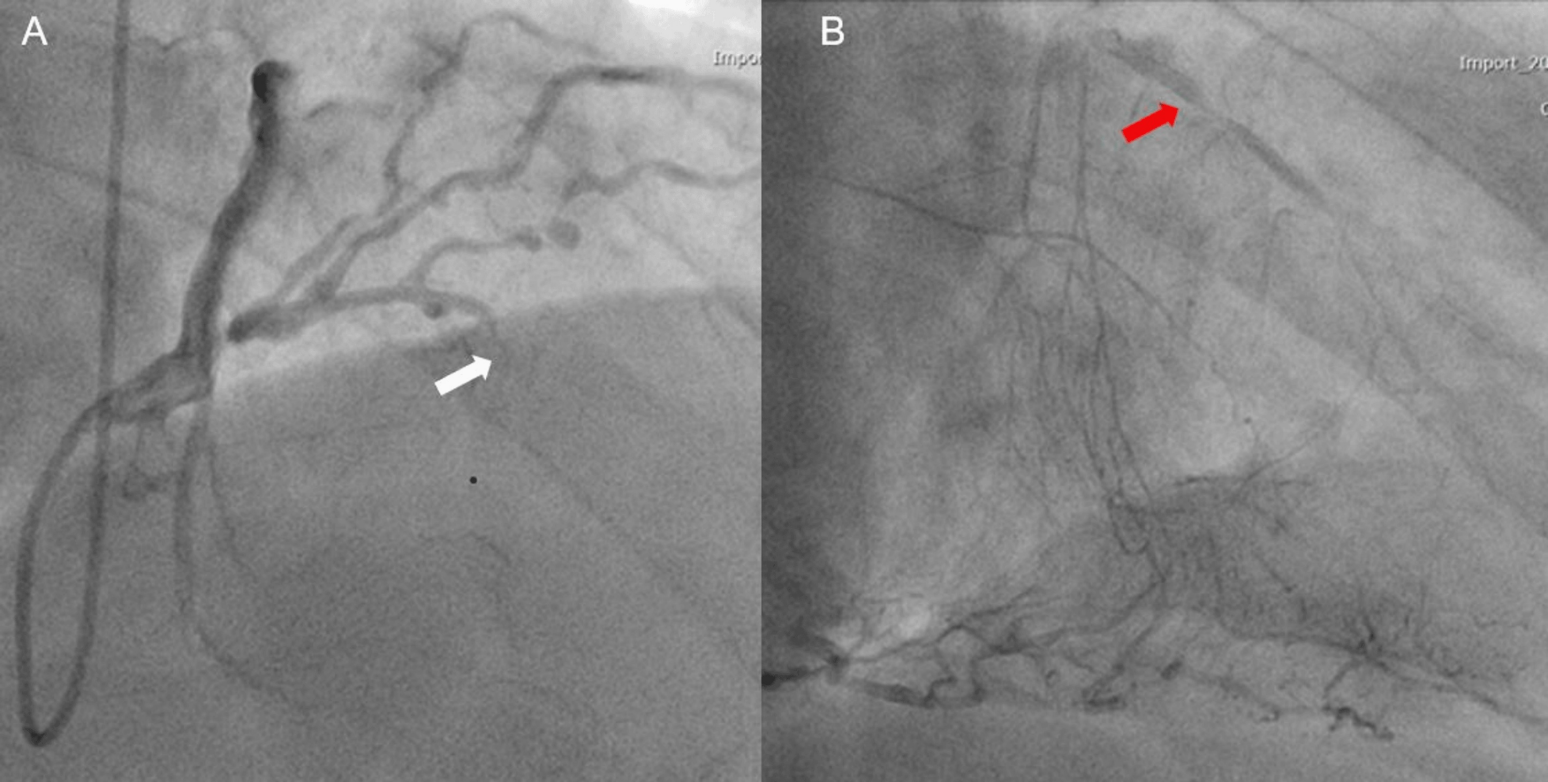
Preoperative cardiac catheterization revealed 99% occlusion (white arrow) of the left anterior descending (A) with collateral flow (red arrow) from the right coronary artery (B)

Preoperative cardiac catheterization revealed 99% stenosis of left anterior descending (LAD) segment 7, 75% stenosis of left circumflex segment 13, and 90% stenosis of right coronary artery segment #4PL (Figure 2).

**Figure 3:**
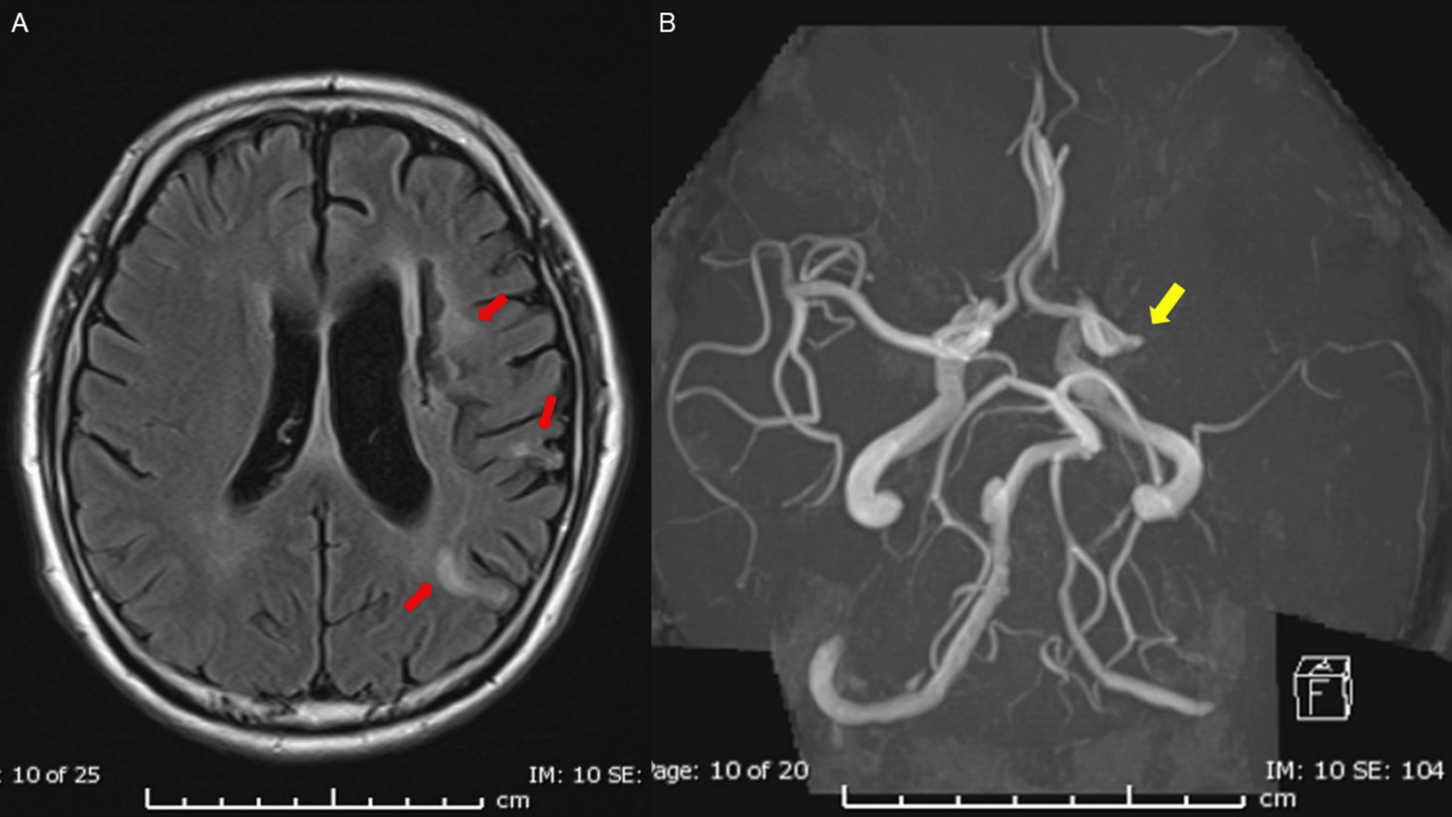
Preoperative head magnetic resonance imaging (MRI) (A) MRI showed an old cerebral infarction in the left cerebrum (red arrows). (B) MR angiography showed occlusion of the left middle cerebral artery (yellow arrow).

Head magnetic resonance imaging (MRI) showed an old cerebral infarction in the left cerebrum and the complete occlusion of the left middle cerebral artery (Figure 3), and the clinical frailty scale was 3.

As a treatment strategy, repair by open TAAA repair was high-risk, and the patient had an extensive aneurysm from the arch aorta, so we decided to use a three-staged surgical hybrid treatment: 1) total arch replacement (TAR) + frozen elephant trunk (FET) technique, 2) TEVAR extension, 3) hybrid TAAA repair (endovascular stent graft and visceral artery reconstruction).

**Figure 4:**
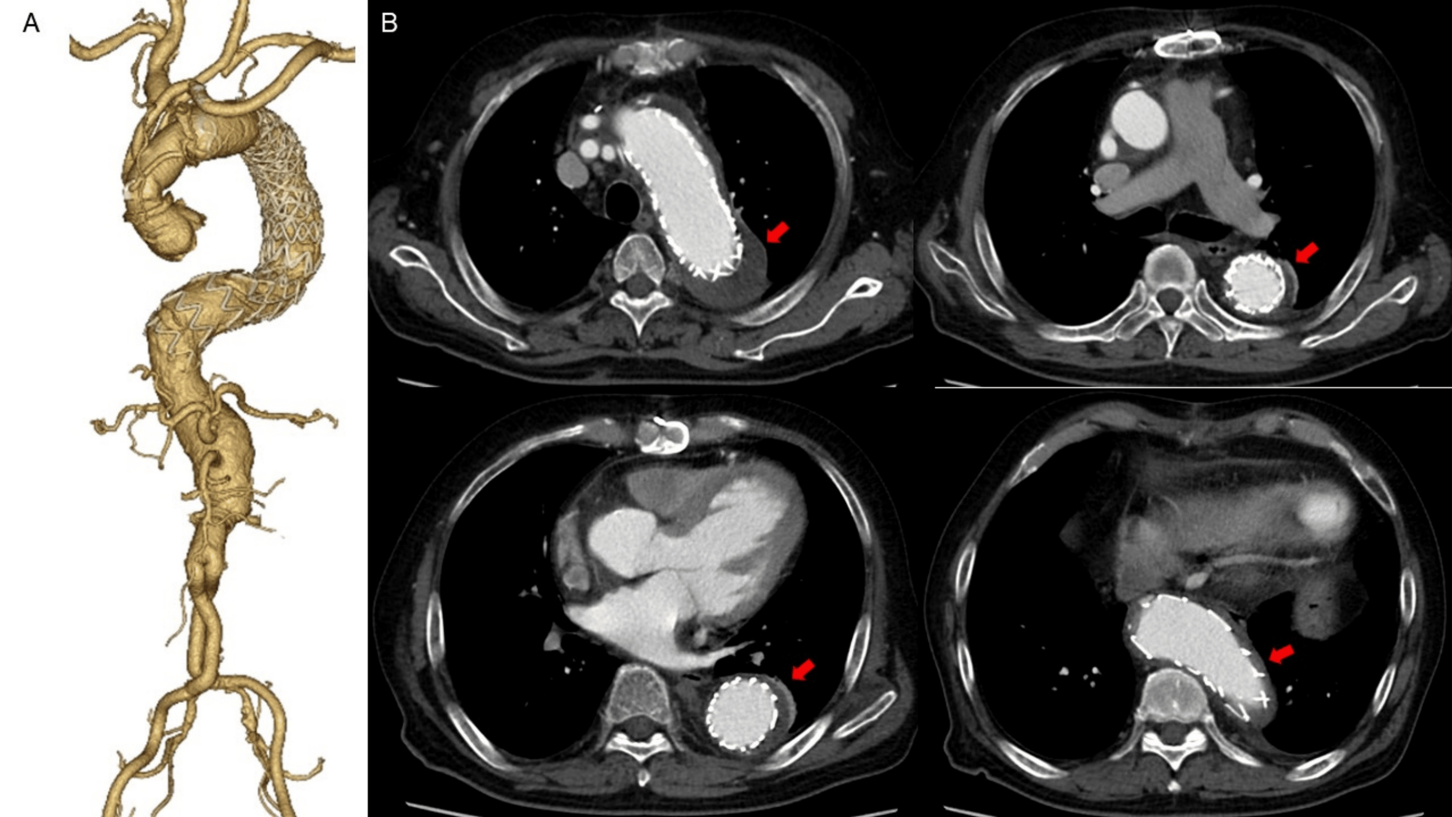
Postoperative aortic contrast-enhanced computed tomography (CT) image showing no endoleak (A) 3D-CT angiography (B) Axial image showed no endoleak (red arrows)

The first operation was performed through a median sternotomy. An 8 mm vascular prosthesis was placed under the left subclavian artery (LSCA). After establishing cardiopulmonary bypass circulation, a no-touch saphenous vein graft (SVG) was anastomosed to LAD. Cooling was started with a target bladder temperature of 28 ℃. After the ascending aorta was clamped, cardiac arrest was induced. Circulation was stopped, and the aortic arch was transected under antegrade selective cerebral perfusion. The LSCA orifice was ligated. A commercially available FET (J Graft FROZENIX, Japan Lifeline, Tokyo, Japan) was inserted into the descending aorta. After distal anastomosis was performed using a four-branch vascular prosthesis, blood was pumped from the lateral branch of the artificial vessel to resume circulation. After the reconstruction of the left common carotid artery, proximal aortic anastomosis was performed, including proximal anastomosis of no-touch SVG. After cardiac beating, the brachiocephalic artery was reconstructed and an 8 mm vascular prosthesis was guided to the mediastinum. Postoperative contrast-enhanced CT showed no endoleak in the aortic arch, and the bypass graft was also patent. Postoperative echocardiography showed that the LVEF improved from 20% to 34%. The second operation was performed one month after the first operation because the patient had undergone abdominal aortic replacement and a one-stage descending thoracoabdominal replacement would risk spinal cord ischemia (SCI). The second operation was extended by TEVAR up to the celiac artery and was a transition from Crawford type III to type IV. A 42x225 mm Zenith Alpha™ proximal device (Cook Medical, Bloomington, Indiana, US) was deployed from the middle of the FET, and then a 46x233 mm Zenith Alpha™ proximal device was deployed 3 cm above the celiac artery. Postoperative contrast-enhanced CT showed no endoleak (Figure 4).

**Figure 5:**
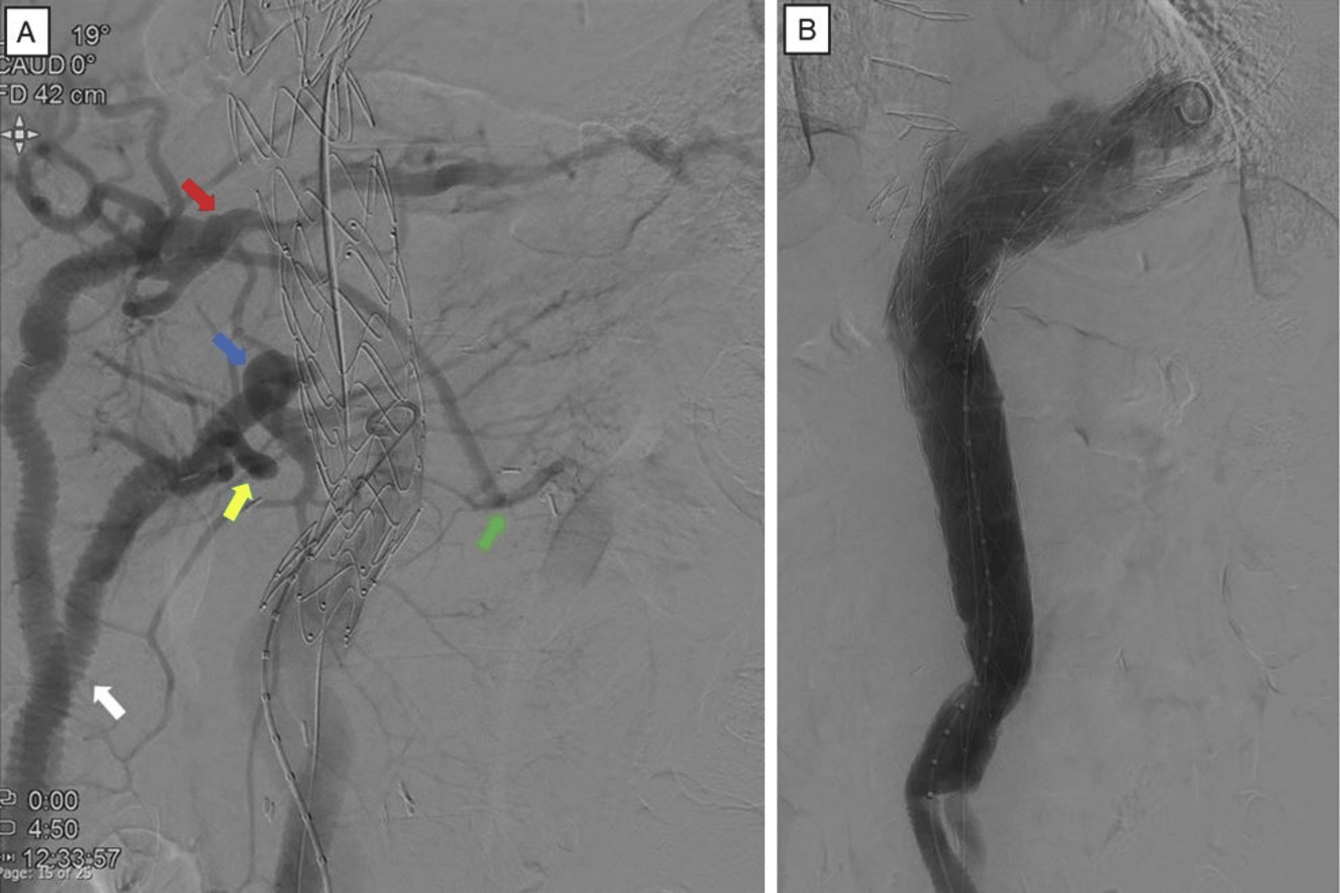
Intraoperative angiography shows visceral arteries reconstruction (A) and stent grafting with no endoleak in hybrid thoracoabdominal aneurysm repair (B) (A) A J-graft 14x7 mm (white arrow) was anastomosed to the superior mesenteric (blue arrow) and proper hepatic arteries (red arrow), respectively, and anastomosed to the right (yellow arrow) and left (green arrow) renal arteries using a saphenous vein graft from each artificial vascular leg.

Thereafter, the TAAA diameter was followed strictly on an outpatient basis. The third operation was abdominal debranching by reopening the abdomen one year after the first operation. A J-graft 14x7mm was anastomosed to the right external iliac artery, which was anastomosed to the superior mesenteric and proper hepatic arteries, respectively, and anastomosed to the right and left renal arteries using SVG from each artificial vascular leg. The origins of each ventral partial branch were ligated during bypass. Approximately two months later, an additional stent-graft insertion (Zenith Alpha™ PT 42x38x173 mm + DE 34x112 mm + PT 32x28x178 mm + DE 30x108 mm) was performed. Hybrid TAAA repair was completed without endoleak (Figure 5), and total aortic replacement was completed. After surgery, the patient was discharged home with no deterioration of renal function and no paraplegia, although it took time to manage his cardiac insufficiency.

## Discussion

This case report demonstrates that a three-staged hybrid TAAA repair can achieve excellent results in managing extensive TAAAs in a very high-risk patient. Open repair of extensive TAAAs remains the gold standard, providing definitive therapy. However, open repair carries significant risks, including mortality and end-organ damage, such as spinal cord ischemia (SCI), pulmonary complications, and renal failure [6]. In particular, Crawford Extent II TAAAs are associated with a higher incidence of mortality, paraplegia, renal failure, and pulmonary complications compared to Extent III or IV TAAAs. Coselli et al. reported a mortality rate of 19% for Extent II TAAAs, almost double the 10% rate for Extent IV TAAAs [7]. On the other hand, some studies suggest that staged hybrid TAAA repair, including TEVAR, reduces mortality and the incidence of SCI [8-10]. However, such cases are relatively rare in routine clinical practice.

There is considerable variability in the techniques, extent of replacement, and duration of staged hybrid TAAA repair. The most frequently reported approach involves proximal TEVAR followed by distal open TAAA repair through a thoracoabdominal incision [8-10]. However, not all patients with Crawford Extent II TAAAs have adequate distal landing zones for TEVAR. Yoshida et al. reported staged hybrid repairs in four patients who initially underwent TAR [11]. These cases, however, were often unexpected TAAA repairs, occurring more than 10 years after initial TAR or associated with conditions such as the rapid expansion of ulcer-like projections caused by type B aortic dissection after TAR or graft infection. Gkremoutis et al. reported the outcomes of 15 cases where TAR using FET was performed first, followed by hybrid treatment with abdominal debranching and stent placement for TAAA [12]. However, one case of permanent paraplegia occurred after TAAA repair, and one patient died due to an aneurysm rupture. This report has described a two-stage procedure; however, the second-stage TEVAR included in our case was not performed. Nakanishi et al. have reported favorable outcomes in two cases of Marfan syndrome, where they performed the TAR with the Bentall procedure and thoracoabdominal aortic replacement, followed by bridging TEVAR [13]. A planned three-stage hybrid replacement from the ascending aorta to address TAAA as seen in the present case, may be relatively rare.

In this case, primary open surgical repair of the Crawford Extent II TAAA was avoided due to the enlarged distal arch at the proximal anastomosis and the high risk of respiratory complications and paraplegia from an extensive thoracoabdominal approach. Surgical treatment of a slowly expanding TAAA with a maximum diameter of 55 mm remains controversial in patients with high surgical risk. Here, the distal arch was also enlarged, and the proximal landing zone for TEVAR was deemed inappropriate. Although the case did not strictly meet the criteria for surgical treatment of the ascending aorta and aortic arch, based on updated aortic treatment guidelines [14], TAR with FET repair was initially performed to establish a proximal landing zone while the risk of rupture remained low. The patient was subsequently converted from Crawford Extent II to Extent IV through TEVAR extension within one month. The final stage of hybrid TAAA repair was carried out one year later, after close follow-up to monitor aneurysm diameter, activities of daily living, and renal function. As Crawford et al. noted, untreated TAAAs have an estimated 76% mortality rate after two years, with half of the deaths caused by aortic rupture [2]. Given the poor prognosis of untreated TAAAs, the staged approach was justified, allowing time to address the patient’s complex and extensive aneurysms within a long-term treatment plan.

SCI remains a significant challenge following TAAA repairs. Various strategies to reduce SCI incidence include cerebrospinal fluid (CSF) drainage, blood pressure augmentation, and staged repairs [15,16]. In this case, CSF drainage was not employed due to the patient’s ischemic heart disease, the need for perioperative antiplatelet therapy, and a history of left ventricular thrombus requiring anticoagulation. Although SCI incidence following TEVAR is reported to range from 3% to 10% [17], staged repair was prioritized to mitigate this risk. The sequential sacrifice of arteries perfusing the spinal cord may promote collateral vessel development, improving spinal cord perfusion. King et al. reported that staged aortic repair had a similar SCI risk to non-staged repair but showed a trend toward reduced permanent SCI risk in the staged repair group [17]. Adopting the Modified COPS Protocol proposed by Tanaka et al. [15], circulation was actively managed and no SCI occurred throughout the process.

Postoperative heart failure was a notable complication in this case. The patient had poor cardiac function and underwent total aortic replacement with artificial vascular grafts and stent-grafts, raising concerns about increased afterload. The elasticity of the aorta is essential for reducing left ventricular pulsatile load, and this elasticity is diminished when the aorta is replaced with inflexible grafts [18]. Yamashita et al. found that descending aortic endografts minimally affected left ventricular contractility in normal canine hearts but increased cardiac afterload and left ventricular hypertrophy [19]. In this patient, postoperative heart failure was carefully managed with blood pressure and fluid control.

This case highlights the effectiveness of planned multistage treatment and the importance of individualized approaches for managing complex and extensive TAAA in high-risk patients. Furthermore, a hybrid treatment combining open surgery and stent graft implantation is suggested as a potentially valuable method to reduce risks. In the future, large-scale clinical studies will be required to evaluate the long-term efficacy and safety of such approaches.

## Conclusions

In this case, we report a staged hybrid TAAA repair with good results in a high-risk patient with various poor prognostic factors such as low cardiac function and chronic renal disease. Extensive thoracoabdominal aortic surgery is still a procedure with high mortality and complications, but if it is not performed, the prognosis is poor. We believe that TAAA repair is feasible in high-risk patients if surgical and perioperative strategies are developed with careful consideration of the patient's risk factors.
